# Radiosurgery for the treatment of dominant hemisphere periventricular heterotopia and intractable epilepsy in a series of three patients^☆^

**DOI:** 10.1016/j.ebcr.2012.10.004

**Published:** 2012-11-07

**Authors:** Chengyuan Wu, Michael R. Sperling, Steven M. Falowski, Ameet V. Chitale, Maria Werner-Wasik, James J. Evans, David W. Andrews, Ashwini D. Sharan

**Affiliations:** a Thomas Jefferson University Hospitals, Department of Neurological Surgery, 909 Walnut Street, Third Floor, Philadelphia, PA, USA; b Thomas Jefferson University, Department of Neurology, Philadelphia, PA, USA; c Thomas Jefferson University, Department of Neurological Surgery, Philadelphia, PA, USA; d Thomas Jefferson University, Department of Radiation Oncology, Philadelphia, PA, USA

**Keywords:** Periventricular nodular heterotopia (PVH), Epilepsy, Stereotactic radiosurgery (SRS), Radionecrosis

## Abstract

Periventricular heterotopia (PVH) is a neuronal migration disorder characterized by masses of gray matter located along the lateral ventricles that commonly cause epilepsy. The benefit of surgical resection of the PVH has been demonstrated in case reports to date; however, the location of the PVH in the paratrigonal region of the lateral ventricles can present significant surgical challenges. Noninvasive modalities of ablating this epileptogenic focus must therefore be considered. We present a small series of three patients who underwent stereotactic radiosurgery (SRS) for inoperable unilateral dominant hemisphere PVHs in order to illustrate the potential benefits and risks of this treatment modality. A total dose of 37.5–65 Gy resulted in seizure freedom for at least 14 months at the time of their last follow-up, even in patients harboring a second independent epileptic focus. Whether intracranial electrode recording truly offers added value is therefore uncertain. The two patients who received higher radiation doses suffered from symptomatic radiation necrosis and associated cerebral edema, requiring further medical intervention, and persistent monocular visual loss in one patient. While a longer interval prior to re-treatment may have been attempted, neither patient demonstrated radiographic findings typically associated with seizure remission. Refractory epilepsy due to PVH may be successfully treated with radiation therapy; but further work is needed to define the optimal dosing parameters in order to lower toxicity to normal tissue.

## 1 Introduction

Periventricular heterotopia (PVH) is a neuronal migration disorder characterized by masses of gray matter located along the lateral ventricles in varying distribution. Although the clinical symptoms in these patients can vary, the most common presentation of PVH cases is epilepsy [1], [2], [3]. Clinical and electrographic findings pointing to a temporal lobe focus in PVH patients have been misleading and have consequently generated poor surgical results in patients who have undergone temporal lobectomy [4]. While more recent studies have shown improved surgical outcome following more specific localization of the epileptogenic focus, no studies have shown the use of stereotactic radiosurgery (SRS) as an alternative to surgical resection [5]. Here, we present a small series of three patients who underwent SRS for unilateral dominant hemisphere PVH. All had been deemed inoperable due to risks of causing aphasia. These cases illustrate the potential benefits and risks of this treatment modality.

## 2 Materials and methods

A retrospective chart review was performed on three patients who presented to our institution with medically-refractory epilepsy secondary to a dominant hemisphere PVH. Patients were treated with SRS between 2003 and 2009 by a multidisciplinary team consisting of epileptologists, radiation oncologists, and neurosurgeons. If a patient continued to experience seizures after a single treatment, a consensus decision was made to administer a second dose of radiation, resulting in a total dose range of 37.5–65 Gy in this series. Follow-up continued until 2011, allowing seizure and complication rates to be recorded over periods ranging from 33 to 46 months.

## 3 Case presentations

### 3.1 Case 1

#### 3.1.1 Seizure history

A 25-year-old right-handed female with no preexisting epilepsy risk factors and no significant past medical history began experiencing complex partial seizures at age 14. Her seizures were preceded by an aura that she described as a “feeling of fading away" or “feeling like blacking out and feeling confused.” Witnessed episodes consisted of lip smacking, chewing, and oral automatisms accompanied by an impaired level of consciousness with an inability to talk or follow commands. Seizures occurred once to twice each week. Medical treatment with carbamazepine, levetiracetam, phenobarbital, and oxcarbazepine failed to control seizures.

#### 3.1.2 Evaluation

**Fig. 1:**
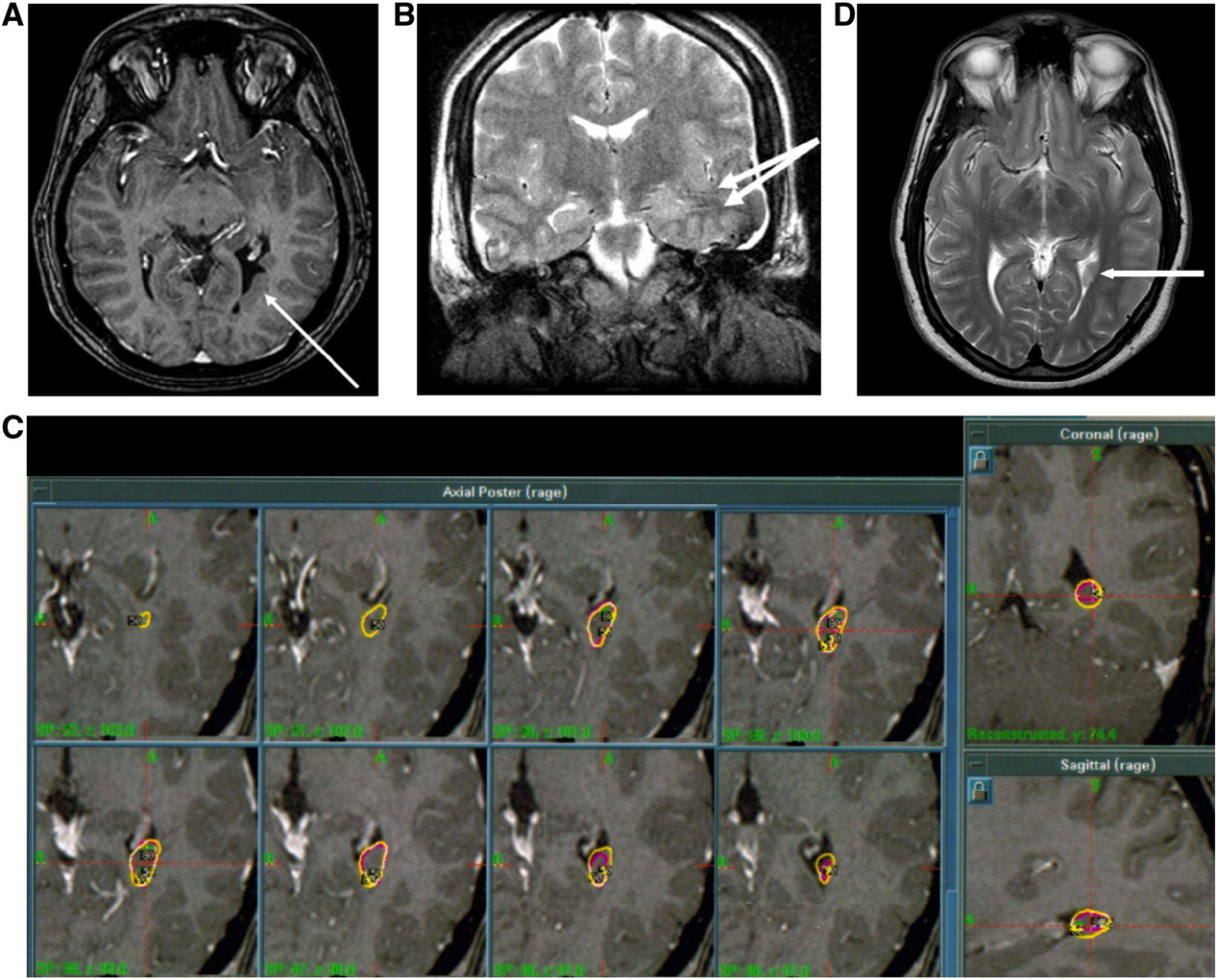
A: T1 MRI demonstrating PVH in the left trigone. B: T2 Coronal MRI status-post implantation of intracranial electrodes. C: Dose plan for Case 1. D: Axial T2 MRI showing left trigone PVH.

Scalp video-EEG monitoring demonstrated left midtemporal interictal sharp waves, and seizures began focally with left sphenoidal sharp waves followed by postictal slowing in the left hemisphere. On neuropsychological testing, she demonstrated a verbal IQ of 102, performance IQ of 104, full scale IQ of 104 and Boston naming score of 44. Additionally, she had weakness in word knowledge, impaired verbal learning, working memory, and facial memory. MRI demonstrated an 8-mm periventricular heterotopia adjacent to the left trigone and associated left mesial temporal sclerosis (Fig. 1A). PET scan showed decreased metabolic activity in the left inferior temporal lobe.

The patient underwent invasive monitoring. Depth electrodes were implanted into the parahippocampus, anterior, middle, and posterior hippocampus; a depth electrode was placed in the heterotopia as well. Four strip electrodes were placed over temporal neocortex (Fig. 1B). Video-EEG recordings revealed seizures that began in the heterotopia and then spread to the hippocampus within 10 s. Interictal spikes were also evident in the heterotopia, hippocampus and temporal basal cortex.

#### 3.1.3 Treatment and follow-up

Because of its location and the inherent risks of surgical resection in the dominant posterior temporal lobe, Gamma Knife Radiosurgery (GKR) was recommended. The first treatment occurred two months after intracranial electrode placement. A dose of 30 Gy was delivered to a 50% isodose line with three shots (Fig. 1C). Unfortunately, she continued to experience seizures at 12-month follow-up and an MRI revealed no lesional changes in the PVH (Fig. 1D). Her seizure activity had changed from one seizure per week to a cluster of 2–9 seizures within 1–2 days that occurred every 2–3 weeks. Therefore, the patient was treated with additional GKR 15 months after the initial treatment. A dose of 35 Gy was prescribed to a 50% isodose line with three shots to a lesion volume of 0.27 cc. The patient has been seizure free for 78 months since the second treatment and stopped medication one year after the second GKR treatment. No complications were noted either clinically or in post-treatment scans.

### 3.2 Case 2

#### 3.2.1 Seizure history

A 26-year-old female began experiencing tonic-clonic seizures at age 21. Her risk factors for epilepsy included premature birth at 35 weeks gestation, learning disability, and mild mental retardation. Her seizures were initially well controlled on phenytoin; but unfortunately, eight months after initial medical treatment she began having complex partial seizures preceded by an aura of dizziness. Zonisamide, lamotrigine, oxcarbazepine, and levetiracetam all failed to control her seizures. Her longest seizure-free interval was 17 days with these medications.

#### 3.2.2 Evaluation

**Fig. 2:**
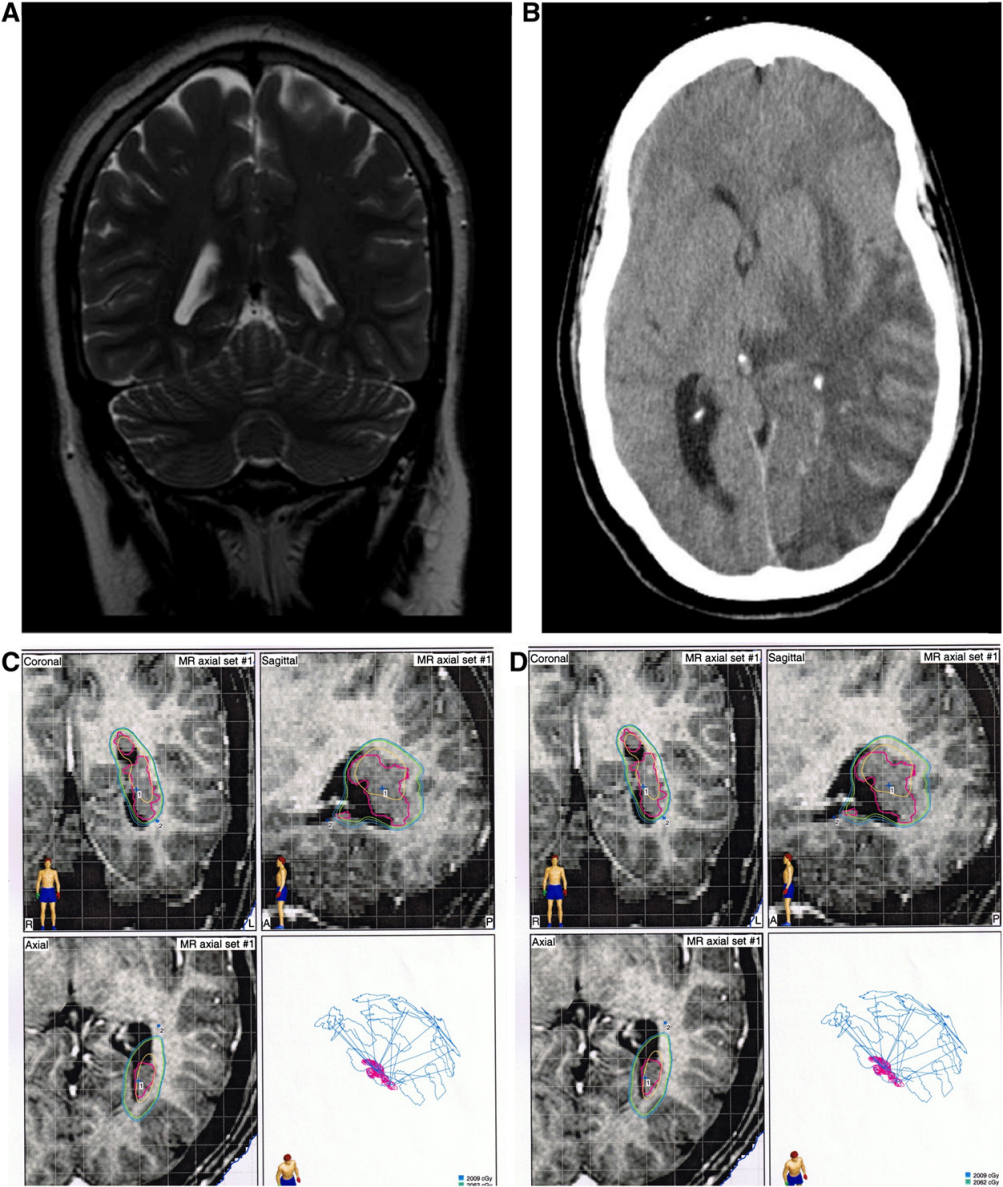
A: Coronal T2 MRI showing left trigone PVH. B: CT scan demonstrating cerebral edema from radiation necrosis. C: Dose plan for initial LINAC treatment for Case 2. D: Dose plan for second LINAC treatment for Case 2.

Scalp video-EEG demonstrated a left sphenoidal onset of seizures with 5- to 6-Hz activity and left sphenoidal interictal sharp waves. Brain MRI revealed a PVH adjacent to the trigone of the left lateral ventricle and left mesial temporal sclerosis (Fig. 2A). Depth electrodes were placed in the left hippocampus and heterotopia; left temporal subdural strip electrodes were placed as well. Video-EEG monitoring with these intracranial electrodes revealed independent seizure onsets zones in the heterotopia as well as in the left temporal neocortex. Depending on the site of onset, ictal spread occurred within 1 s between these two regions. Interictal spikes in the heterotopia, left hippocampus, and temporal neocortex were also noted.

#### 3.2.3 Treatment and follow-up

Due to the location of the heterotopia and the risks of surgical resection, linear accelerator (LINAC) radiosurgery to the heterotopias was recommended. A dedicated stereotactic LINAC (BrainLab Inc, Munich, Germany) was used for treatment with dynamic conformal arcs methodology. The first treatment occurred 8 months after invasive monitoring and consisted of a dose of 21 Gy delivered to a 92% isodose line, covering 98% of the lesion (Fig. 2C). Seizures continued and she began to experience an increased seizure frequency. Follow-up imaging demonstrated no radiation effect and the patient therefore underwent a second treatment session 10 months after initial treatment. This time, 40 Gy was administered to a 91% isodose line covering 100% of the lesion — measuring 2.21 cc (Fig. 2D). Seizure frequency decreased over the next 6 months with a three-month seizure-free period. She was maintained on pre-treatment doses of oxcarbazepine and levetiracetam.

Her post-treatment course was then complicated by intermittent headaches, a mild expressive aphasia, facial edema, papilledema, and slight hyperesthesia in the left V2 distribution that began 7 months following the second LINAC treatment. A CT scan of her brain demonstrated left-sided cerebral edema, for which she was placed on steroids (Fig. 2B). MRI revealed a ring-enhancing lesion consistent with radionecrosis. As she was initially weaned from her steroids, she experienced a recurrence of her symptoms and required hospitalization and reinstitution of a steroid regimen. She had visual deterioration to acuity of 20/70 and optic nerve sheath fenestration was performed. After recovering from this second episode, she has been seizure free for 54 months at the time of last follow-up. Vision completely recovered in one eye and remained impaired in the other.

### 3.3 Case 3

#### 3.3.1 Seizure history

A 27-year-old right-handed male with no seizure risk factors developed complex partial seizures at age 20. He characterized these seizures as starting with an aura of dizziness with ensuing loss of awareness and confusion. During these episodes, he spoke intelligently but did not follow commands or recall the events. He reported clusters of 3 to 4 seizures per week, which occurred approximately every 2 months. At the time of admission, his seizures had been shown to be refractory to prior treatment with oxcarbazepine and was taking both topiramate and levetiracetam.

#### 3.3.2 Evaluation

**Fig. 3:**
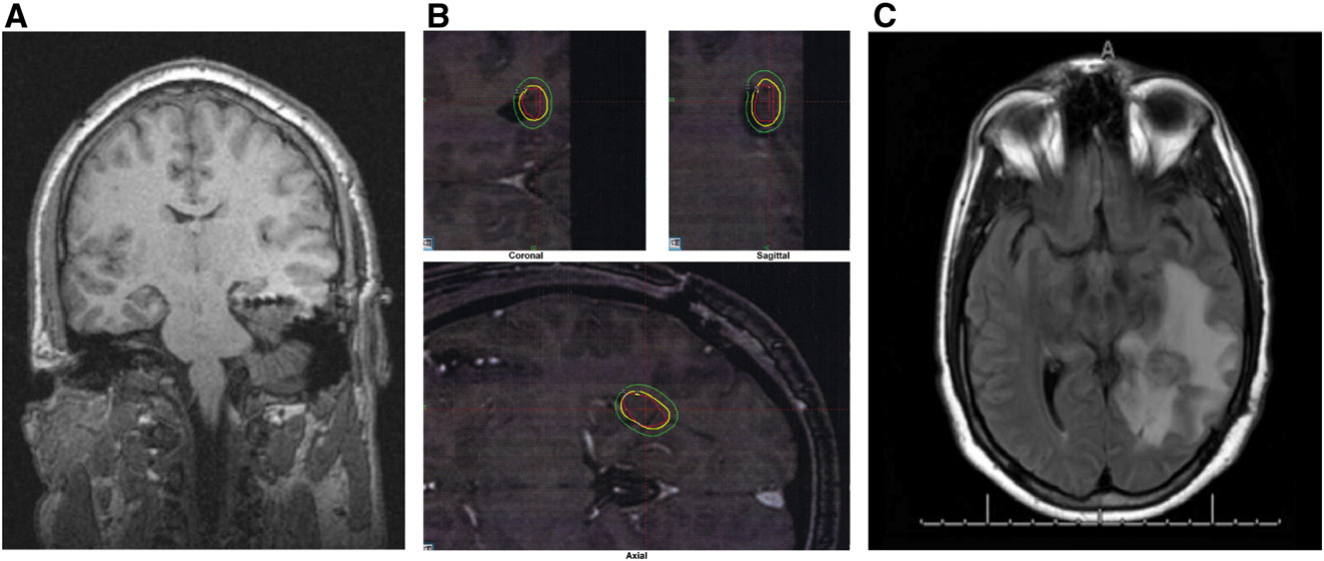
A: Post-operative coronal T1 MRI showing depth electrodes. B: Dose plan for Case 3. C: Axial FLAIR MRI at 19 months post-treatment showing radiation necrosis.

Scalp video-EEG demonstrated frequent interictal left temporal lobe slowing and sharp waves. Complex partial seizures began with 8-Hz activity originating at T5. Imaging studies included an MRI consistent with PVH involving the left lateral ventricle in the trigone and region of the occipital horn (Fig. 3A).

Intracranial video-EEG confirmed that the heterotopia was the location of seizure onset for some seizures, but also demonstrated some seizures appeared to start simultaneously in the overlying temporal lobe neocortex. Similarly, the interictal EEG demonstrated spikes in the heterotopia, neocortex and hippocampus.

#### 3.3.3 Treatment and follow-up

As with the prior two cases, it was decided that the best modality of treatment was SRS. Two months after localization via invasive monitoring, he was treated with 37.5 Gy GKR delivered to a 50% isodose line, covering 100% of the lesion, which measured 0.5 cc in volume (Fig. 3B). He had no seizures for the first six months after treatment, at which time his seizures recurred at a rate of 1–3 seizures per month.

Nineteen months after GKR treatment, he developed severe headaches without a focal neurologic deficit. A CT scan and subsequent MRI demonstrated lateralized cerebral edema consistent with radiation necrosis (Fig. 3C). He was treated with a course of steroids and maintained on topiramate and levetiracetam. At the time of last follow-up, approximately 33 months after his treatment, he had been seizure free for 14 months and continued to take topiramate and levetiracetam.

## 4 Discussion

While structural features in patients with PVH may vary, they share a central theme of drug-resistant epilepsy. Clinical presentations and surface EEG pointing to mesial temporal lobe origins have been demonstrated to be misleading, and traditional temporal lobe resection for these patients has resulted in poor outcomes. Such results are most likely due to incorrect localization — with seizures actually originating from the PVH [5], [6]. Improper localization may stem from the connections of the PVH to surrounding allocortex and neocortex, which amplifies its epileptic activity and leads to temporal neocortical and hippocampal involvement in interictal spikes and seizures [5], [6].

Further evidence of the importance of diagnosing a PVH is the benefit shown in case reports of surgical resection of the lesion. Aghakhani described a good outcome (Engel Class ID) in a patient with an epileptogenic focus confined to the occipital neocortex who underwent a transcortical occipital approach for the removal of two nodules and a small rim of overlying cortex. Although a focal lesion remained at a five-year follow-up, the patient required only a single antiepileptic drug. Scherer et al. similarly reported a patient with a PVH adjacent to the posterior horn of the left lateral ventricle, who underwent a resection of this lesion and subsequently achieved seizure freedom for 2 years [7]. In both cases, depth electrodes localized seizure onset to the PVH and not in the hippocampus. These results align with the current philosophy of epilepsy surgery — that good outcomes occur when a single seizure generator can be localized and resected [8], [9], [10].

In two of our patients, however, invasive monitoring indicated two independent zones of ictal onset: the PVH and the temporal lobe neocortex. While our suspicion of the PVH as the primary seizure generator led us to target that lesion, we also planned for the possibility of a later cortical resection. Dosing plans were restricted to the PVH alone and did not expose the relatively distant secondary ictal onset zones to any significant radiation (Figs. 1C, 2C, D, 3B). It is therefore highly unlikely that any clinical effects may be attributed to the inadvertent involvement of those separate seizure onset zones in the treatment plan. These results contrast the aforementioned literature regarding the importance of a single focus of epileptogenesis, since treating the PVH alone was effective in this small series.

The location of a PVH can present significant surgical challenges with increased morbidity and mortality in patients [6]. Unilateral PVH is frequently located in the paratrigonal region of the lateral ventricles [10], where surgical resection is challenging due to intervening and adjacent eloquent cortex — particularly in the dominant hemisphere. For this reason, different modalities for ablating this epileptogenic focus must be considered.

All three of our patients harbored a unilateral left dominant PVH that was identified as a seizure onset zone. Therefore, in light of the success of SRS in patients with epilepsy of other underlying etiologies [3], [11], [12], [13], [14], [15], [16], [17], [18], [19], [20], [21], [22], [23], [24], [25], [26], [27], [28], [29], [30], [31], [32], this less invasive modality was chosen. Patients with epilepsy attributable to hypothalamic hamartomas have successfully undergone radiosurgery with dosages of 12–20 Gy [12], [13]. Similarly, Regis reported a 60% seizure-free outcome with > 5 year follow-up in 9 of 16 patients who underwent 24 Gy of GKR for mesial temporal lobe epilepsy (MTLE) [3]. Although doses ranging from 18 to 25 Gy have been reported in the treatment of MTLE [3], [15], [16], [17], [18], [19], [20], [21], [22], [23], [24], [25], [26], [27], [28], [29], [30], [31], [32], doses of 20 Gy and 24 Gy have been extensively studied, with long-term outcomes of > 60% seizure freedom associated with both dosages [17], [18], [29]. We are well aware that the dosages presented here are significantly higher than those reported in the literature to date. The first two patients received comparable initial radiation doses of 21–30 Gy; but when this treatment failed to provide adequate seizure control, re-treatment increased the total dose exposure to 61–65 Gy. While Case 1 demonstrated a favorable outcome after 65 Gy of exposure, Case 2 suffered significant morbidity from 61 Gy. In light of the complications encountered in Case 2, the initial radiation dose was reduced to 37.5 Gy for Case 3 (Table 1).

**Table 1:** Summary of treatment courses for 3 patients with periventricular nodular heterotopia.

Patient	Location of seizure focus	Volume [cc]	SRS modality	Initial dose [Gy]	Time before recurrence [months]	Retreatment time interval [months]	Retreatment dose [Gy]	Seizure outcome	Cerebral edema
Case 1	Heterotopia	0.27	GKR	30.0	–	15	35.0	Class I	No
Case 2	Heterotopia & left temporal lobe	2.21	LINAC	21.0	1	10	40.0	Class I	Yes
Case 3	Heterotopia & left temporal lobe	0.50	GKR	37.5	6	–	–	Class I	Yes

Indeed, higher doses of radiation have been associated with complications secondary to radiation necrosis and associated cerebral edema [24]. In his experience with 23 patients, Regis reported four cases of visual field defects and three cases of morbidity requiring corticosteroids for radiation-induced changes [11], [14], [15]. While corticosteroids are typically sufficient to reduce such edema [14], [15], [16], [17], [31], some patients have required surgical decompression [20]. Unfortunately, one of our patients shared a similar fate, requiring optic nerve sheath fenestration and suffering from a permanent monocular visual deficit.

Although Case 3 also demonstrated radiographic findings of radiation necrosis and associated cerebral edema (Fig. 3C), this finding only manifested clinically as headaches. In fact, noticeable radiation changes may be necessary to achieve a therapeutic effect. Chang et al. reported that the appearance of vasogenic edema 9–12 months after treatment correlated with the onset of seizure remission [29]. Similarly, Rheims et al. noted that subnecrotic radiation-induced tissue changes might contribute to the antiepileptic effect of GKR. The optimal dose should therefore induce these necessary changes while avoiding adverse clinical symptoms.

Finding this therapeutic range for radiation to a PVH is complicated by the inability to use MRI alone to assess the effects of treatment. Radiation-induced changes in MRI have been found to vary even when doses have been standardized [29]. Therefore, the use of proton (^1^H) magnetic resonance spectroscopy (MRS) may be helpful in further characterizing the specific effects of radiation. As with MRI, radiographic changes in MRS have been reported to peak approximately one year after radiation [32]. Specifically, radiation necrosis has been characterized by a decrease in N-acetylaspartate (NAA), creatine (Cr), and choline (Cho) peaks; but an increase in the lactate peak [31–34]. Furthermore, the severity of injury has been associated with greater reductions of N-acetylaspartate (NAA) than creatine (Cr), which itself has declined more than choline (Cho). This results in low NAA/Cho and NAA/Cr ratios, but a high Cho/Cr ratio [31], [32], [33]. In addition, higher lactate/Cr ratios have been associated with more extensive mass effect changes [34]. Further work must be performed in order to determine if specific values can be correlated with therapeutic effects. Unfortunately, since none of our patients underwent MRS after radiation, we are unable to help in this endeavor.

Given the small sample size of three patients in this series, recommending an ideal dose of radiation remains difficult. Furthermore, the outcomes of the first two patients after a single dose is unclear, as re-treatment after 10 and 15 months was likely premature. Current experience with MTLE patients suggests that seizure cessation is usually delayed, with a response latency of at least 6 months and stabilization after 24 months [3], [11], [14], [15], [16], [17], [18], [24]. Therefore, while one could argue that we should have waited longer before re-treating both of these patients, neither showed any of the aforementioned radiographic effects of radiation at 10 and 12 months. Taking such factors onto consideration, we have determined that the initial 30 Gy was likely ineffective for the first patient. At the same time, the 37.5 Gy dose in Case 3 led to seizure freedom at the cost of symptomatic headaches requiring corticosteroids. Therefore, the appropriate dosage may lie between 30 and 37.5 Gy. Once again, a significant amount of work must be completed to first find the ideal dosage and subsequently to test the efficacy of that dose.

## 5 Conclusion

Refractory epilepsy due to PVH may be successfully treated with radiation therapy. These cases also suggest that intracranial electrode recording does not provide any added value in patients with a PVH. As long as the lesion was ablated, demonstrating an independent zone of seizure onset in the temporal lobe neocortex did not preclude seizure freedom. While the clinical response to SRS for epilepsy has a reported latency of approximately 1–2 years, the importance of radiation-induced vasogenic edema and role of MRS have also been highlighted. While the upper limit of radiation is likely less than 37.5 Gy, further work is needed to define the optimal total dose and to explore the possible application of fractionated radiotherapy as a means to lower radiation toxicity to surrounding normal tissue.
